# Unexpected malocclusion in a 13,000-Year-old Late Pleistocene young woman from Mexico

**DOI:** 10.1038/s41598-022-07941-7

**Published:** 2022-03-07

**Authors:** José Rubén Herrera-Atoche, James C. Chatters, Andrea Cucina

**Affiliations:** 1 Department of Orthodontics, School of Dentistry, Autonomous University of Yucatan, Calle 61-A No. 492-A Costado Sur del Parque de la Paz por Avenida Itzáes, Col. Centro, 97000 Mérida, Yucatán México; 2 Applied PaleoScience, Washington, USA; 3 School of Anthropological Sciences, Autonomous University of Yucatan, Carretera Merida-Tizimin, Tramo Cholul, 97305 Mérida, Yucatán Mexico

**Keywords:** Anthropology, Oral anatomy, Evolutionary developmental biology

## Abstract

To analyze the etiological factors behind the malocclusion of a Late Pleistocene woman (named Naia), who is the best-preserved of the earliest individuals of the American continent. The examination of Naia’s malocclusion was performed through cephalometric and occlusal analyses, and by measuring her mandible. Her data were then compared to published data for modern, medieval, and postmedieval samples and seven Late Pleistocene individuals. Naia presented her permanent dentition fully erupted, except for the impacted mandibular third molars. She presented a class II molar malocclusion with crowding. The dental widths and mandible measurements were similar to or smaller than modern standards. The degree of dental wear was light. The cephalometric analysis confirmed a skeletal class II relationship, with a retrusive mandible and protruded upper incisors. Naia’s mild level of dental wear is consistent with a low masticatory force, in a time when the norm was a high amount of grinding. The low masticatory forces help explain Naia’s small jaws and crowding. However, it does not clarify Angle’s class II relationship. Naia is an example that environmental factors are insufficient to explain the onset of malocclusions and emphasizes the importance of understanding hereditary factors’ role.

## Introduction

In 2007 a group of divers who were mapping the underwater Outland Cave, a segment of the Sac Aktun cave system in Quintana Roo, Mexico, made a remarkable finding; 600 m away from their insertion point, they found a vast subterranean chamber 62 m in diameter with its bottom at a maximum depth of 55 m below sea level. They called it the “Hoyo Negro” (Black Hole)^1^. In their subsequent immersions, divers found the skeleton of a human being on the bottom of Hoyo Negro, surrounded by skeletons of extinct fauna^2^. After proper analysis, it was determined that the subject was a 15 to 17 years old female who lived 10,976 ± 20 radiocarbon years ago^3^ or between 12,970 and 12,770 calibrated years ago, in the Late Pleistocene period^4^. She was named “Naia” after a Greek water nymph.

**Figure 1:**
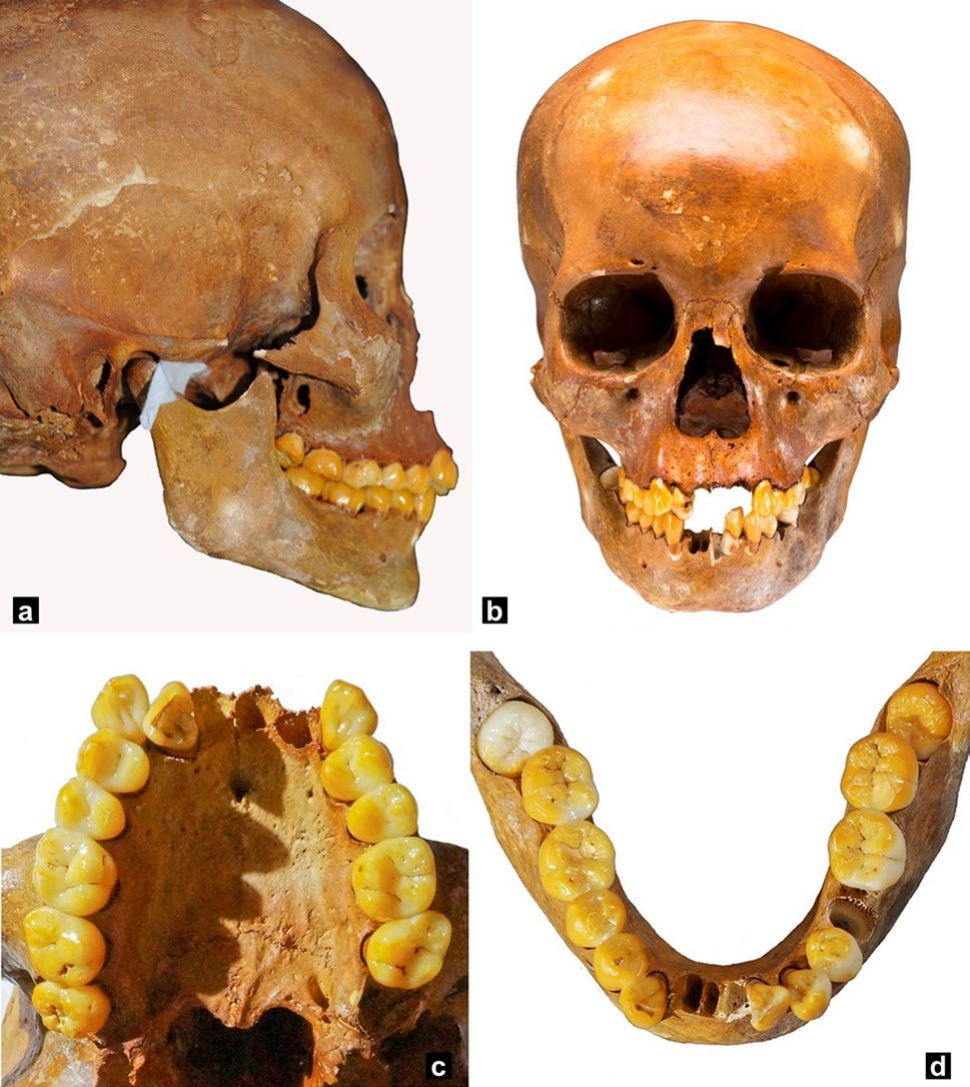
(**a**) Lateral view of the skull. (**b**) Frontal view of the skull. (**c**) Upper occlusal view. (**d**) Lower occlusal view (photos taken by A. Cucina).

Since Naia is one of the oldest individuals of the American continent and so well preserved, she has caught the scientific community's interest in many fields. Regarding her oral health, Naia had an anomalously high (for the epoch) amount of dental caries and acute periodontal disease (probably because she had a soft diet including fruits). It has also been reported that she had a very low degree of occlusal wear and an Angle class II malocclusion with severe crowding^5^ (Fig. 1).

This finding was exceptional since malocclusion is considered a modern condition that is a consequence of our way of life. The literature demonstrates that malocclusion prevalence has increased after the introduction of agriculture and then again following the industrial revolution^6^. Therefore, the presence of such complicated malocclusion is quite unexpected in a Late Pleistocene individual. This study aims to analyze the etiological factors behind Naia's malocclusion within the Late Pleistocene/Early Holocene context and compare it with modern standards.

## Materials and methods

This study is part of the “Proyecto Aqueólogico Subacuático Hoyo Negro” (Hoyo Negro Underwater Archaeological Project), which is an official undertaking of Instituto Nacional de Antropología e Historia (National institute or Archaeology and History/Mexico). The research protocol was approved, and permission granted by the Consejo de Arqueología (Mexican Archaeology Council) of the Instituto Nacional de Antropología e Historia.

The study of Naia's malocclusion was carried out by conducting occlusal analysis, mandibular measurement, and cephalometric analysis. Her data were then compared to published data for medieval and postmedieval samples, and to gonial angle and wear from seven Late Pleistocene female individuals from North and South America. Gonial angle and wear were scored by RH and AC based on published images, except for Peñon III, whose data are reported by Jiménez et al.^7^.

### Occlusal analysis, mandibular measurements and dental wear

Dental crowding and alignment were assessed (Fig. 1). Using a Mitutoyo digital caliper (Mitutoyo®), the canine, premolar, and molar widths and mandibular measurements were calculated (Table 1). To evaluate the occlusion, an orthodontist positioned the mandibular condyles in their respective glenoid fossae (with appropriate spacing at the temporomandibular joint), and the jaws were articulated in dental occlusion using the evidence of dental wear as a guide^8^ (Fig. 1).

**Table 1:** Definitions of occlusal and mandibular measurements.

Occlusal measurements	Definition
**Upper arch**	
Maxillary intercanine width	Distance between upper canines' cusp tips
Maxillary first premolar width I	Distance between upper first premolars' buccal cusp tips
Maxillary first premolar width II	Distance between upper first premolars' central fossae
Maxillary second premolar width I	Distance between upper second premolars' buccal cusp tips
Maxillary second premolar width II	Distance between upper second premolars' central fossae
Maxillary intermolar width I	Distance between upper first molars' mesiobuccal cusp tips
Maxillary intermolar width II	Distance between upper first molars' central fossae
**Lower arch**	
Mandibular intercanine width	Distance between lower canines' cusp tips
Mandibular first premolar width I	Distance between lower first premolars' buccal cusp tips
Mandibular first premolar width II	Distance between lower first premolars' central fossae
Mandibular intermolar width I	Distance between lower first molars' mesiobuccal cusp tips
Mandibular intermolar width II	Distance between lower molars' central fossae

Mandibular measurements	Definition
Mandibular body length	Distance between gonion and menton
Ramus height I	Distance between gonion and condyle
Ramus height II	Distance between gonion and coronoid
Intercondylar width	Distance from the external face of one condyle to the other
Intercoronoid width	Distance from the apex of one coronoid process to the other
Gonial angle	Angle formed by the intersection of the inferior border of themandible and the posterior border of the ramus

To assess occlusal dental wear, the authors followed the methodology described for Naia in Cucina et al.^5^, which ranges from grade 1 to 8 based on the degree of dentine exposure. Grade 1 codes for no wear (i.e., the enamel crown is pristine), while grade 8 represents the complete loss of the crown with the root functioning as occlusal surface. Wear was eventually classified as light (grades 2 and 3—wear facets are small, and the possible appearance of dentine is minimal), moderate (grades 4 and 5—dentine patches are moderate, and the appearance of secondary dentine is null or still very slight) or severe (grades 6 to 8—severe patches of dentine and clear evidence of secondary dentine).

### Cephalometric analysis

Naia’s skeletal segments had been previously CT-scanned separately before we could start our morphometric and morphological analyses. No X-rays were taken of the whole skull and mandible in occlusion due to the skeleton’s fragility.

Dolphin Imaging 11.8 software was used to make the cephalometric analysis of the subject. The program was set to use the accepted international norm values for a 16-year-old female without specification of ethnic origins. However, given the differences between a traditional cephalometric analysis performed on a cephalometric x-ray in comparison to a lateral skull image, some analyses deviated from standard procedure, as follows:

1. Only cephalometric measurements with points located outside the skull were used.
2. The soft tissue measurements were eliminated.
3. Given that the subject only has two incisors still in the sockets (upper right and lower left lateral incisors), those were used for the incisor position analysis.

The measurements fulfilling the above criteria are described in Table 2.

**Table 2:** Cephalometric measurements.

Ricketts cephalometric measurements	Definition	Modern norm	Naia
Interincisal angle	(U1-L1)°	130 ± 6	127.3
Upper incisor protrusion	(U1-APg) (mm)	3.5 ± 2.3	7.7*
Lower incisor protrusion	(L1-APg) (mm)	1 ± 2.3	1.9
Upper incisor inclination	(U1-APg)°	28 ± 4	33.2*
Lower incisor inclination	(L1-APg)°	22 ± 4	19.5*
Occlusal plane to Frankfurt	(Occ Plane-FH)°	6.8 ± 5	10.5
Convexity	(A-NaPg) (mm)	0.7 ± 2	6.4**
Mandibular plane inclination	(MP-FH)°	23.9 ± 4.5	25.4
Maxillary depth	(FH-NA)°	90 ± 3	93.3*
Facial axis	(NaBa-PtGn)°	90 ± 3.5	85.8*
Facial angle	(FH-NaPg)°	88.6 ± 3	85.4*
Porion location	(Pr) (mm)	− 38.6 ± 2.2	− 47.2***
Cranial Deflection	(FH-NaBa)°	24.6 ± 3	27.3
Ramus position	(FH-CfXi)°	76 ± 3	63.6***
Lower face height	(ANS-Xi-Pm)°	45 ± 4	42.3

Jarabak cephalometric measurements	Definition	Modern norm	Naia
Gonial/jaw angle	(Ar-Go-Me)°	122.9 ± 6.7	122.1
Mandibular body length	(Go-Gn) (mm)	75.2 ± 4.4	70.8*
Upper gonial angle	(Ar-Go-Na)°	52 ± 7	52.2
Lower gonial angle	(Na-Go-Me)°	71.2 ± 6	69.9
Ramus height	(Ar-Go) (mm)	48.5 ± 4.5	41.5*
ANB	(A-Na-B)°	1.6 ± 1.5	8.5***
IMPA	(L1-MP)°	95 ± 7	96.8
Lower incisor—facial plane	(L1-NaPg) (mm)	1.5 ± 2	5.8**
Upper incisor—facial plane	(U1-NaPg) (mm)	5 ± 2	11.4***
Mandibular plane to occlusal plane	(Occ Plane-MP)°	17.4 ± 5	14.9

°Angles are in degrees.

*Naia’s value falls outside of one standard deviation from the modern norm.

**Naia’s value falls outside of two standard deviations from the modern norm.

***Naia’s value falls outside of three standard deviations from the modern norm.

### Institutional approval

Morphological and morphometric methods used in this study were carried out in accordance with the regulations established by the Mexican Archaeology Council (Consejo de Arqueología), which supervises and authorizes archaeological and bioarchaeological (i.e., ancient human remains) studies.

We state that no experiments have been carried out on the skeletal remains of Naia, but only morphological and morphometric analyses.

Last, this study is part of the Proyecto Aqueológico Subacuático Hoyo Negro, which is an official undertaking of the Instituto Nacional de Antropología e Historia. Access to the skeletal remains and permissions for the morphological and morphometric analyses, were granted by the Mexican Archaeology Council, which waived the informed consent statement.

## Results

Naia presented her permanent dentition fully erupted, except for the mandibular third molars that were tilted forward and impacted. She presented an Angle class II molar malocclusion and a sharp curve of Spee. Despite the lack of three incisors in each jaw, it was possible to determine anterior crowding in both arches with the upper right lateral in a crossbite relationship.

**Figure 2:**
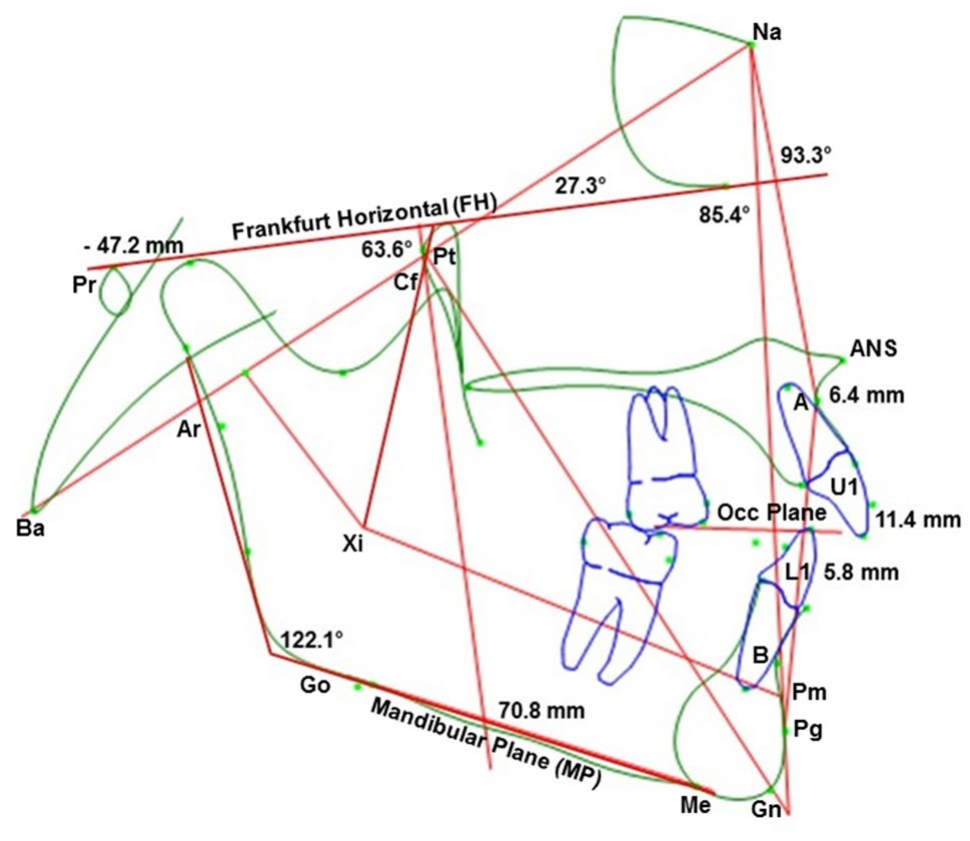
Cephalometric analysis (U1: Upper incisor; L1: Lower incisor; A: A point; Pg: Pogonion; Na: Nasion; Ba: Basion; Pt: Pterygoid point; Gn: Gnathion; Pr: Porion; Cf: Center of face; Xi: Xi Point; ANS: Anterior Nasal Spine; Pm: Protuberance menti; Ar: Articulare; Go: Gonion; Me: Menton; B: B point).

The results of the cephalometric analysis (Fig. 2) are shown in Table 2 in comparison with the expected norm values for modern populations. As we can appreciate, of the 25 measurements, Naia falls outside one standard deviation from the norm in eight cases (represented by one asterisk next to Naia’s measurement), in two cases she falls outside two standard deviations (two asterisks next to her value), and in four more cases three standard deviations (three asterisks next to her values). The measurements of the interdental widths are shown in Table 3^9–16^, while mandibular measurements are shown in Table 4.

**Table 3:** Comparison of Naia’s interdental widths with other reported populations.

Year	Naia	Normando^16^				Bălan^13^	Sayin^9^	Uysal^14^	Lombardo^10^	Oliva^11^	Forster^12^	Alkadhi^15^
	2022	2016				2014	2004	2005	2013	2018	2008	2018
Population sample	N/A	Xicrin-Kaiapó	Arara-Laranjal	Arara-Iriri	Assurini	Romania	Turkey	Turkey	Systematic Review	Italy	USA	Saudi Arabia
Angle class/sex	CII Div 1/female	N/A	N/A	N/A	N/A	CII Div 1/M&F	CII Div 1/M&F	CII Div 1/M&F	CII Div 1/M&F	CI/Females	NA/Females	CI/Females
**Upper arch**												
Maxillary intercanine width	33.87	36.04*	36.1*	37.73*	38.78*	33.9	33.56	34	33.59^^^	32.66	32.15	33.71
Maxillary first premolar width I	38.16					39.9	39.46	39.9	39.93^^^		38.68	40.35*
Maxillary first premolar width II	32.33									36.39*		34.28*
Maxillary second premolar width I	46.55						44.32				43.46	44.68*
Maxillary second premolar width II	40.41											38.97*
Maxillary intermolar width I	50.55	53.54*	53.34*	53.67*	56.2*	51.9	50	52.1	50.95^^^		49.03	49.21*
Maxillary intermolar width II	45.88						45.5			44.28	44.16	43.42*
**Lower arch**												
Mandibular intercanine width	26.36	27.55	27.56	28.14*	29.08*	27.9	26.8	27.9	29.08^^^	25.45	24.11*	26.02*
Mandibular first premolar width I	33.94					34.7	34.58	34.8	38.34^^^		31.95	33.79
Mandibular first premolar width II	29.52									29.91		30.57*
Mandibular intermolar width I	44.93	45.23	45.32	45.26	47.65*	46.2	43.7	46.8	49.98^^^		42.52	43.67*
Mandibular intermolar width II	41.48									41.17	39.22	39.51*

*Outside the standard deviation. ^^^Standard deviation not included in the article. (CII Div 1) Class II Division 1. (CI) Class I. (M&F) Males and Females.

Superscript numbers refer to the bibliographic source of measurements.

**Table 4:** Naia’s mandibular measurements compared with mean values for medieval and post-medieval Europe^6^.

Mandibular measurements	Naia	Rando^6^	
		Medieval	Post-medieval
Mandibular body length	72.76	73.77 ± 11.16	79.03 ± 5.29*
Ramus height I	50.71	66.36 ± 4.11*	62.79 ± 4.63*
Ramus height II	48.17		
Intercondylar width	110.44	117.05 ± 3.88*	111.64 ± 6.71
Intercoronoid width	91.15		
Gonial angle	125	122.5 ± 6.15^^^	127.98 ± 8.08^^^

*Outside the standard deviation. ^^^The authors published the value of the angle above 90°. Superscript numbers refer to the bibliographic source of measurements.

Naia presented a marked skeletal class II relationship of the jaws according to both cephalometric analyses (Ricketts’ convexity and Jarabak’s ANB). This anteroposterior discrepancy was due to a combination of traits; on the one hand, the upper maxilla is in a forward position (maxillary depth), while the mandible is in a backward location (facial angle). Additionally, the mandibular body is small, and the ramus is in a rearward position.

Regarding the incisors, the upper ones were in a severe protrusive position while for the lower ones there are some variations among the measurements. For example, the lower incisor protrusion and the incisor mandibular plane angle (IMPA) values are within the norm. However, one measure shows them in a retroclined position (lower incisor inclination), and the lower incisor/facial plane even places them in a protrusive location.

Last, Table 5 lists the gonial angle and degree of occlusal wear in seven Late Pleistocene female individuals in comparison with Naia^7,17–22^.

**Table 5:** Comparative measurements of gonial angle with other Late Pleistocene and Early Holocene female individuals from North and South America.

Individual	Dating (Ka)	Age (years)	Gonial angle	Wear
Naia^5^	12.9	15–17	125	Light
Peñon III^7^	12.7	24–26	106	Moderate/severe
Buhl^17^	12.6	17–21	113	Severe
Wilson Leonard II^18^	~ 12	20–25	115	Moderate/severe
Horn Shelter 2, B2^22^	10.9	~ 12	130	Light
Gordon Creek^19^	11.2	25–30	116	Moderate/severe
Arch Lake^20^	11.4	17–21	124	Light
Antoniäo Cave^21^	10	20–22	122	Severe

Superscript numbers refer to the bibliographic source of measurements.

Dates are thousands of calibrated years before present (ka).

## Discussion

**Figure 3:**
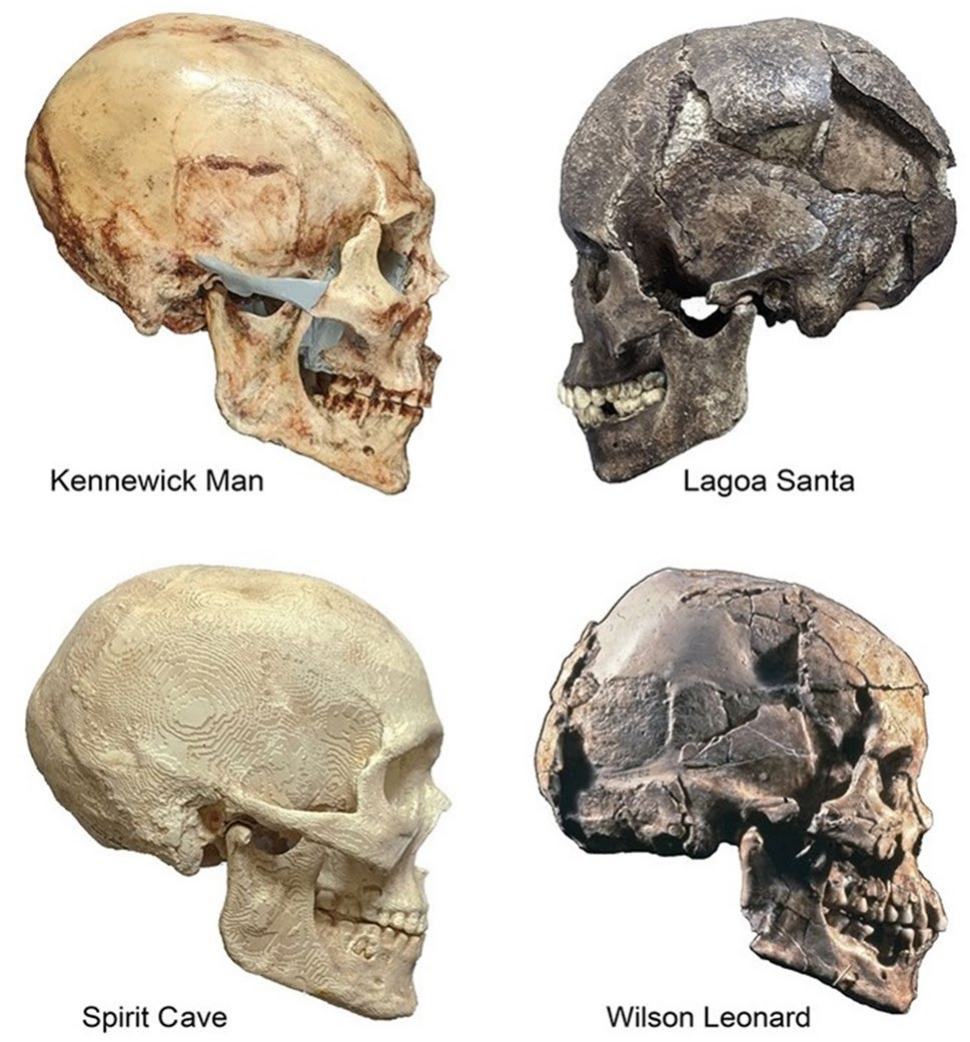
Examples of other Paleoindians (photos taken by J. Chatters). All but the Wilson Leonard individual are males.

Today, it is generally accepted that malocclusion is a condition that has increased its prevalence and severity with the advance of technology^8,23,24^. Evidence in the literature suggests that two crucial moments in history effected the increase of malocclusions; the first was the introduction of agriculture as basic subsistence, and the second was the industrial revolution^6^. In both cases, processed and hence softer food reduced the required masticatory forces, affecting the jaws' growth and development and the amount of grinding of the teeth. Late Pleistocene groups were hunter-gatherers in whom we would expect to find a high amount of grinding^5^. Therefore, Naia’s teeth and hence jaw musculature might be expected to have been used to the same extent as they apparently were in the majority of the coeval individuals recovered so far. However, this is not the case^5^. Compared with other Late Pleistocene/Early Holocene subjects described in the literature, Naia's amount of tooth grinding is mild, which has been attributed to a soft diet without fibrous elements^5^. Moreover, her upper arch presents anterior crowding and protrusion and the lower arch anterior and posterior crowding, which confirm a retarded or reduced growth of her odontostomatognathic structure. Compared with other North and South America individuals of approximately the same period (Fig. 3), it stands out that the norm was a high amount of grinding [see Cucina et al. 2019^5^ for a comparative chart of occlusal wear, and also Table 5] with no crowding, a flat/mild curve of Spee, and proper arch forms^7,17–19,25–28^. None of the individuals that can be inspected based on published photos shows a full-step Class II malocclusion, like the one recorded in Naia^5^. Dental crowding similar to that in Naia can indeed be appreciated also in the 12-Year-old female from Horn Shelter. Unfortunately, the published images^22^ present maxilla and mandible separately, making it impossible to assess her Angle class. The Arch Lake Woman, a female individual slightly older than Naia at about 17–21 years of age, shows a similar amount of occlusal wear^20^, but just a very slight crowding was present in the upper arch. However, given that the anterior section of the mandible is not present, it is not possible to assess whether the lower arch manifested any form of crowding. This evidence shows the paramount importance of lack of masticatory force and teeth grinding to the development of crowding.

An intense masticatory force induces the development of prominent bones and tooth grinding. Concerning the bones' size, recently it has been reported that the size of the maxillary basal bone is related to crowding for both upper and lower arches^29^. Therefore, Naia's maxillary width dimensions (Table 3) might explain crowding in the upper and even in the lower arch. On the other hand, tooth grinding creates broader interproximal surfaces that help avoid slippages between teeth, keeping them aligned^30^. In some cases, the amount of grinding is so extensive, that it reduces the required arch perimeter. This is because the crowns of anterior teeth become narrower closer to the gingival third.

Following the same train of thought, Naia's inter-molar and inter-canine measurements are smaller compared with modern indigenous populations that have kept their ancestral dietary habits^16,31^. Even matched with urban populations with softer diets, Naia's measurement are similar or smaller in many cases (Table 3)^9–16^. Moreover, female individuals reach their final arch width by about 12 years of age^32^, so, given Naia’s estimated age of 15–17 years, it was likely that the width values had already reached their maximum.

As mentioned above, Naia’s mandibular measurements were small compared with modern standards and matched with medieval and postmedieval European populations^6^. This finding is not typical of Late Pleistocene American women, with the exception of the 12-Year-old female from Horn Shelter 2-B2; the few individuals who were nearly coeval with Naia that present intense occlusal wear and still retained most of their teeth at death also exhibit perfectly aligned teeth in their dental arches. This is also true in the even fewer cases, like Arch Lake, where dental crowns had not been worn down excessively. Further, it is interesting to remark that Naia exhibits a gonial angle measurement similar to today's female norm. Studies have shown that, once there is a shift to a softer diet, the gonial angle tends to increase, creating a posterior-rotation of the mandibular body and the long face-form seen in modern individuals with a weak bite force^6^. The comparison with the other Late Pleistocene female individuals shows that Naia presents the second widest angle, with 125 degrees, exceeded only by the Horn Shelter^22^ child with 130 degrees. Arch Lake^20^ presents a similar value, with 124 degrees, followed by Antoniäo Cave^21^ with 122 degrees; all the others fall well below 120 degrees. Interestingly, the three individuals with a wider, more open gonial angle (Horn shelter, Naia and Arch lake) are the ones that manifest the lowest degree of occlusal wear, stressing the concept that masticatory forces contribute to shape this part of the mandible.

Cephalometric analyses indicate Naia had a small and retrognathic mandible, which is frequent in modern class II individuals^33^. The literature points out that class II malocclusion was rare in ancient times^23,24,34^, and that it increased in modern populations. This fact leads again to the theory that reduced mastication forces resulting from a soft diet are largely responsible. However, although this theory offers a reasonable explanation for some of Naia's malocclusion traits such as crowding or irregularity, it does not account for other aspects of it, like the Angle's class II molar relationship. The retrognathic position is not easy to explain without considering other elements such as the growth of the cranial base^35^ and the position of the glenoid fossae^36^, which affect the sagittal position of the mandible.

The cephalometric analysis shows that cranial deflection is within the normal range; however, Naia's portion and ramus position are retrognathic, and logic leads to the conclusion that one is a consequence of the other. In this case, the explanation is that the glenoid fossae are retrognathic, directly impacting Naia's class II development.

Although some environmental factors are considered^24^, and malocclusion etiology is multifactorial, studies that have addressed this issue consider that there is also a significant hereditary component to the development of a class II retrognathia^37,38^, and parents pass it on to their offspring^39^. In 2014 Moreno-Uribe et al. described five phenotypes for class II malocclusion^40^, of which Naia would be consistent with their cluster 5 since she has a maxillary protrusion, mandibular retrusion, deep overbite, and increased overjet. In particular, a polygenic inheritance with incomplete penetrance and variable expressivity has been proposed for class II division 1^41^.

Recent genetic research has emphasized the relevance of the hereditary component for the etiology of malocclusions and recommends that orthodontists consider it before choosing a treatment option to help ensure success. Understanding that genes have a significant impact on the presence of specific malocclusions will lead to more precise treatment alternatives^41^. Naia is an example of the necessity for further research in this field since environmental factors are no doubt important, yet still insufficient to explain her malocclusion.

## Conclusions

The analysis of Naia’s complex malocclusion leads to the conclusion that, although rare, cases of class II traits are older than commonly accepted. They have been present since ancient times, although they did occur at much smaller rates in the past. The same factors that contribute to creating malocclusion today were also present among some hunter-gatherer populations 13,000 years ago. Results also highlight the importance of reduced masticatory force and teeth grinding to the onset of modern malocclusions and emphasize the importance of further understanding the role of hereditary factors.

## Data Availability

All data generated or analyzed during this study are included in this published article.
